# Impact through versatility: Patterns of *in vivo* dosimetry utilization with TLD across a large multi-site radiotherapy department

**DOI:** 10.3389/fonc.2022.918178

**Published:** 2022-10-18

**Authors:** Peta Lonski, Tomas Kron

**Affiliations:** ^1^ Department of Physical Sciences, Peter MacCallum Cancer Centre, Melbourne, VIC, Australia; ^2^ Sir Peter MacCallum Institute of Oncology, University of Melbourne, Melbourne, VIC, Australia; ^3^ Centre for Medical Radiation Physics, University of Wollongong, Wollongong, NSW, Australia

**Keywords:** patient safety, *in vivo* dosimetry, Radiotherapy, TLD (thermoluminescent dosimeters), quality assurance

## Abstract

The complexity of modern radiotherapy treatment pathways necessitate input from different professions to ensure treatment is delivered safely and as planned. *In vivo* dosimetry is one method of treatment verification providing the opportunity for both in-field verification or out-of-field measurements. It was the aim of this work to review the impact of an *in vivo* dosimetry programme with t.he view to justify resources and assist in developing a plan for equipment acquisition. Results of 310 (approximately 2 per 1000 treatment fractions) *in vivo* measurements were reviewed over a two-year time span. The *in vivo* dosimetry programme using thermoluminescence (TLD) chips was able to detect three significant treatment errors, amongst some 13 000 patients treated. These errors would likely to have been undetected through other quality assurance measures. Increasing demands in workload were found to be associated with commissioning of new equipment and techniques. A skilled operator with knowledge of TLD physics, treatment planning system (TPS) dose calculation algorithms and radiation transport proved to be essential for appropriate interpretation of TLD results particularly in complex radiation delivery scenarios. TLD continues to play a large role in patient safety and quality assurance at our institution.

## Introduction

Modern radiotherapy treatment pathways are complex processes involving several steps with input from numerous professions, the timely cohesion of which is vital to ensure treatment is delivered safely and as planned. Quality control measures are used to prevent errors from propagating along the treatment process. Modern technology provides a degree of automation, reducing the risk of random errors though potentially increasing the risk of systematic errors (1). *In vivo* dosimetry is often used in addition to other quality assurance measures as a final confirmation of the radiation dose which is delivered to a patient. While in recent literature *in vivo* dosimetry is often largely equated to electronic portal image-based methods (2, 3) some applications such as out-of-field dose, skin dose measurements and dose reporting for critical structures, require different detectors.

The International Atomic Energy Agency (IAEA) states in report (No. 8) of the Human Health series in 2013 that “a recent series of radiotherapy accidents in advanced radiotherapy centers led to the conclusion that only the direct measurement of the dose actually delivered to the patient gives the information as to whether the treatment was carried out as it was intended”, subsequently recommending *in vivo* dosimetry for “routine verification of the dose delivery for all groups of patients undergoing radiotherapy” (4). This statement supports previous recommendations going as far back as 1976 when the International Commission on Radiation Units and Measurements stated in Report 24 that “an ultimate check of the actual treatment given can only be made by using *in vivo* dosimetry” (5). This recommendation could arguably apply to radiotherapy technologies of the time; however, more recent publications have served to further support this notion. A recent survey of *in vivo* dosimetry practices across the UK (6) revealed that 73% of respondents to the survey perform *in vivo* dosimetry. Of those who responded to the survey, 5 (< 4%) reported identifying any errors through their *in vivo* dosimetry programme. Errors included incorrect focus to skin distance, field size or beam energy, missing compensators and manual calculation errors. The authors conclude that *in vivo* dosimetry may prevent serious radiation accidents. Current and future *in vivo* dosimetry techniques are discussed by Mijnheer et al. (7), who recommend that curative treatments should all be verified through *in vivo* dosimetry and argue that the methods need to evolve to be precise and appropriate in highly modulated modern radiotherapy treatments.

The present work aims to summarize key findings from a review of two years of *in vivo* dosimetry conducted using thermoluminescent dosimetry (TLD) across five campuses of a large radiotherapy department. The aim of the review was to assess demand for measurements, type of work performed, resources required and impact of the measurements in order to inform a long-term business plan to integrate *in vivo* dosimetry in our department. Patient TLD results were collated between May 2015 and July 2017, covering a range of treatment techniques including megavoltage photons and electrons, kilovoltage photons, intensity modulated radiotherapy (IMRT) techniques, total body irradiation (TBI) and total body electron (TBE) treatments.

## Methods and materials

### TLD for *in vivo* dosimetry

Based on long term use of TLDs with LiF : Mg,Ti, a new TLD system for patient *in vivo* dosimetry using LiF : Mg,Cu,P chips (3.1 x 3.1 x 0.9 mm^3^, TLD100H, Harshaw Chemical Company, Solon, OH, USA) was commissioned in early 2015. This system comprises a relative dosimetric measurement technique as described by others (8) whereby a subset of TLDs from a batch are given a known radiation dose under reference conditions using the same radiation quality as the *in vivo* measurement. These chips, referred to as ‘standards’, were read during the same readout cycle as patient TLDs. Ratios of the response of patient TLDs to the standard TLDs which have been given a known dose was used to convert TLD light signal into dose (Gy). Sensitivity factors were used to correct for slight variations in response from individual chips within the TLD batch. This technique can, under controlled conditions, yield 2% precision on a 95% confidence interval for megavoltage radiation. This does not however include other uncertainties associated with *in vivo* dosimetry, such as TLD placement and energy response in the kilovoltage (kV) range (9) resulting in uncertainties for kV measurements typically of the order of 5% of the measured value. For routine TLD measurements at our center a 5% measurement uncertainty for megavoltage treatments of both photons and electrons is stated, while for orthovoltage this increased to 10% to account for energy dependence and steeper dose gradients across the size of a TLD. TLD standards were irradiated in the same beam quality as patient treatment to minimize impact of energy dependence.

Two of the five radiotherapy campuses across our institution are equipped with Harshaw 5500 automated TLD readers. Campuses which are not equipped with a TLD reader have access to the TLD service *via* internal courier, where TLDs are sent out upon request and returned to the host facility for analysis. One campus utilized MOSFETs for *in vivo* dosimetry during the study period and is not included in the analysis. Temperature-time profile settings are identical for both readers and the same readout profile is used for all patient-related TLD measurements. TLD procedures are standardized across campuses. Results of patient TLD measurements across four campuses were collated for this study (Campus 5 used MOSFETs instead of TLDs, results of which are not included herein).

### Patient results and data collection

TLD measurements were normally performed for a single fraction unless a repeat was either requested (for example, in the event of a discrepancy), or if it was standard practice, as in the case of TBE. Upon receiving a patient TLD measurement request a physicist packages the required number of TLDs ensuring they have been annealed and have stable sensitivity factors, defined as less than ± 2% variation in response over three consecutive calibration cycles. Number of TLDs provided, placement of TLDs, and appropriate choice of packaging is decided by the physicist in consultation with radiation therapists (RTs) and radiation oncologists (ROs) as appropriate. The medical physicist (MP) would then either place the TLDs on the patient or provide instructions for the RTs on where to place them. TLD placement was case-specific and depended on the clinical need. For dose verification, for example, TLDs were placed in a homogenous region close to or on the target (where possible) after reviewing the planned dose distribution. For skin reactions TLDs were placed where the reaction was most evident, for TBE treatments TLDs were placed at standard anatomical locations. After TLDs are read out, measured dose is compared to planned dose where possible and results recorded in a spreadsheet as well as reported to the RO *via* the record and verify system (MOSAIQ, Elekta, Stockholm, Sweden), where results are permanently stored with the patients’ radiotherapy records. For TBE, planned doses are not available for comparison and TLD measurements are deemed appropriate or not based on past experience, with our current TBE technique having 30 years’ experience and patient TLD data for reference. In some cases, out-of-field doses are not available due to the region of interest being beyond the extent of the planning CT, for example in the case of pacemakers. For out-of-field measurements treatment planning system (TPS) calculations have known limitations and as such a discrepancy between measured and planned dose would be expected, with the TPS anticipated to under-estimate out-of-field dose. A discrepancy was identified in the difference between measured and expected dose being greater than the measurement uncertainties associated with a particular TLD measurement, typically of the order of 5%.

TLD records for all patients since implementation of the new system were analyzed for this study spanning a two-year period from May 2015 to June 2017.

## Results

### Measured dose

**Figure 1:**
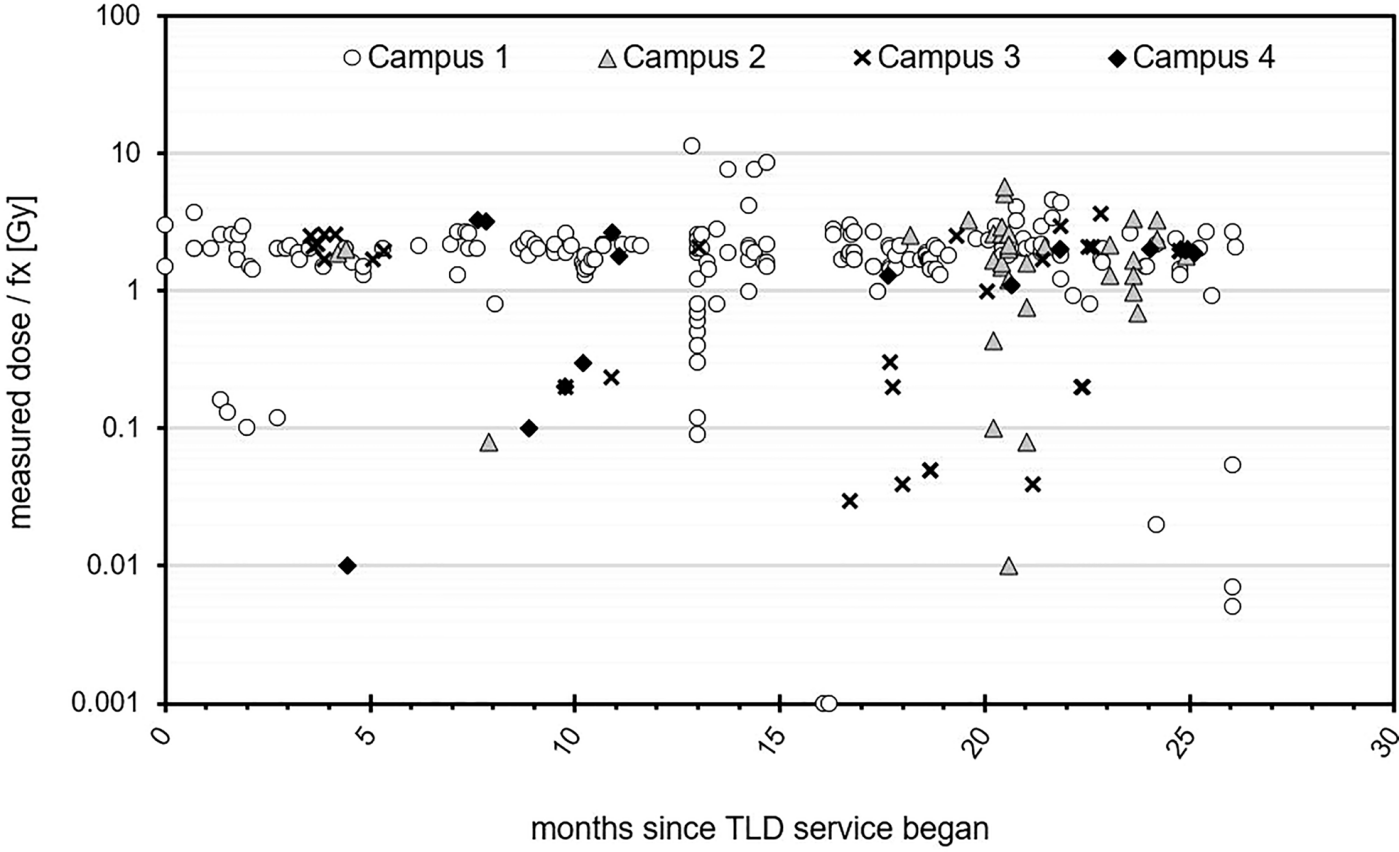
Measured dose per fraction for all patients measured over a two-year time period across four campuses.

Measured dose per fraction for all patients across each campus over the two-year time span are shown in **Figure 1**. A wide dose range is represented as can be seen from the logarithmic scale. This demonstrates the diverse application of TLDs, from hypo-fractionation regimens such as SABR (example skin dose assessment) to lower out-of-field dose measurements, for pregnancy and pacemaker assessments. The dose linearity for TLD-100H is reported elsewhere (10) and is linear in the range of doses reported in this work. The limit of detection for our TLD system has been previously reported elsewhere (11).

Clusters in the figure represent introduction of new techniques or clinical studies and trials. Some higher fractional doses are noted particularly at Campus 1, which were SXRT cases. TLDs were requested in these cases due to the relatively large potential impact of slight setup variations.

### TLD workload

**Figure 2:**
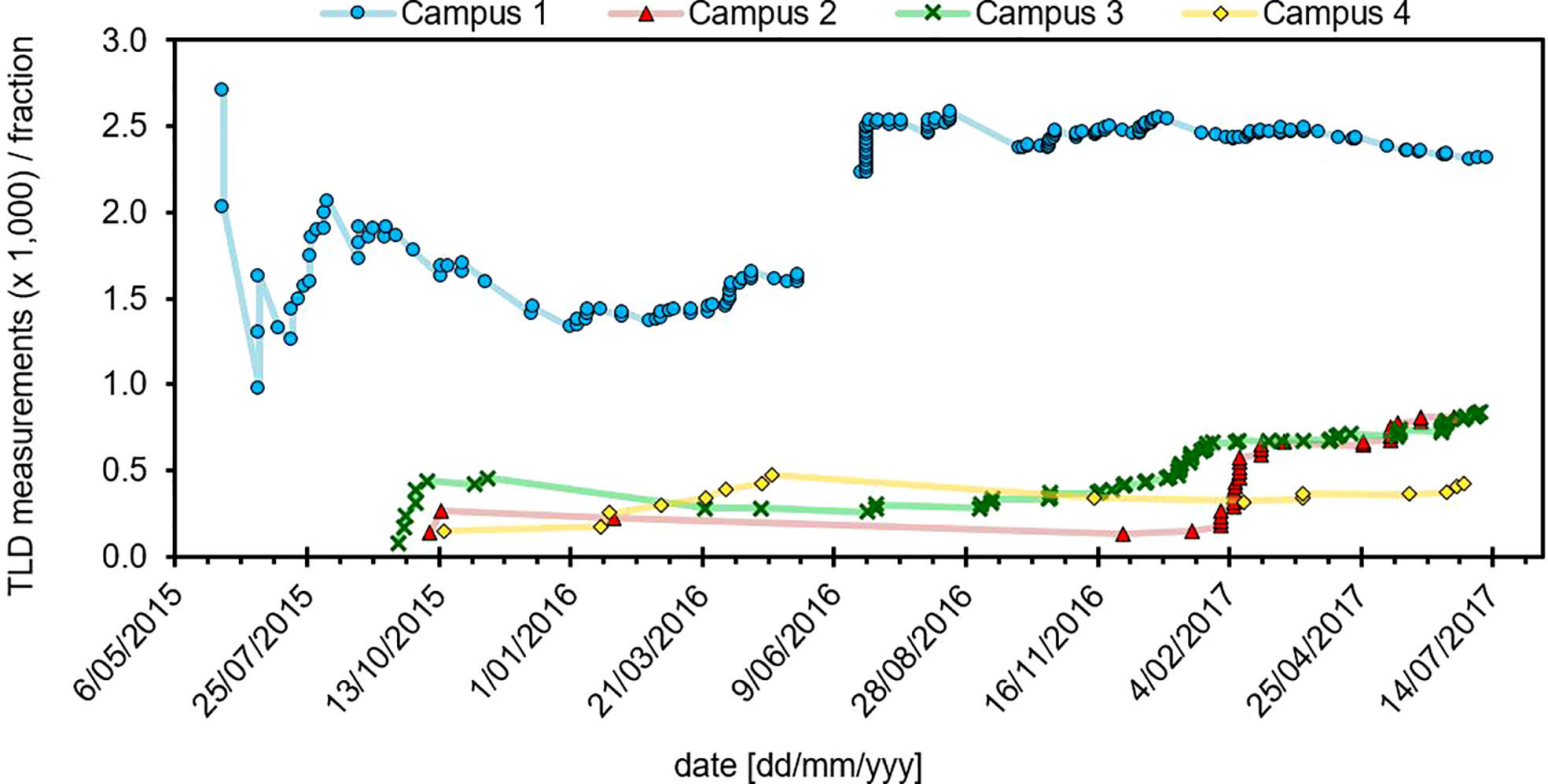
Role of the TLD service across each campus over a two-year time frame. Campus 1 and 2 were relocated to new facilities, which opened in July 2016 and January 2017, respectively.

The number of *in vivo* TLD measurements (x 1,000) per treatment fraction delivered is represented in 255 patients received TLD measurements, for a total of 310 TLD measurements after accounting for repeat measurements for some individual patients. **Figure 2** for each campus. At the time the average number of fractions per treatment course was around 20. Campuses 1 and 3 were equipped with TLD readers, with other campuses having access *via* internal mail delivery. Campus 1 had the highest TLD workload. A jump in TLD work can be seen for Campus 1 in July 2016 and Campus 2 in February 2017. Both of these facilities opened at new sites during these times and TLD measurements were performed for a subset of initial patients, chosen to cover a variety of treatment techniques and modalities across all new machines. An increase in TLD measurements was also noted for Campus 3 in January 2017 at which time this particular campus implemented Eclipse electronic compensator IMRT for breast radiotherapy and performed TLD measurements on all new patients treated with this technique. TLD workload was maintained at all campuses following initial increase.

Campus 1 can be seen to have the highest TLD workload. It has a TLD reader on-site and 6 linear accelerators plus a SXRT unit as well as HDR brachytherapy, making it the largest campus with greatest variety in treatment modalities. It is also the only campus offering TBE and TBI.

### Resources

The costs associated with TLD measurements were estimated for our institution based on a model previously proposed by Kesteloot et al. (12). The initial purchase price of a fully automated TLD reader and oven plus TLDs and associated handling equipment is estimated to be AUD $190,000 (2018 Australian pricing). Assuming 20 years of service for these items the annual fixed cost is AUD $22,000 using the model described by Kesteloot, which considers the fixed and variable costs associated with TLD and provides a model which calculates costs based on patient workload and initial setup expenses. With a TLD workload of 200 measurements per year, and assuming 2 hours of physicist time for TLD preparation, readout and analysis, the cost per *in vivo* measurement is here estimated to be AUD $200 based on the model provided by Kesteloot, though this will vary if other professional craft groups are involved in TLD placement and handling. This cost is reduced if the number of measurements increases across the organization.

### Measurement applications

**Figure 3:**
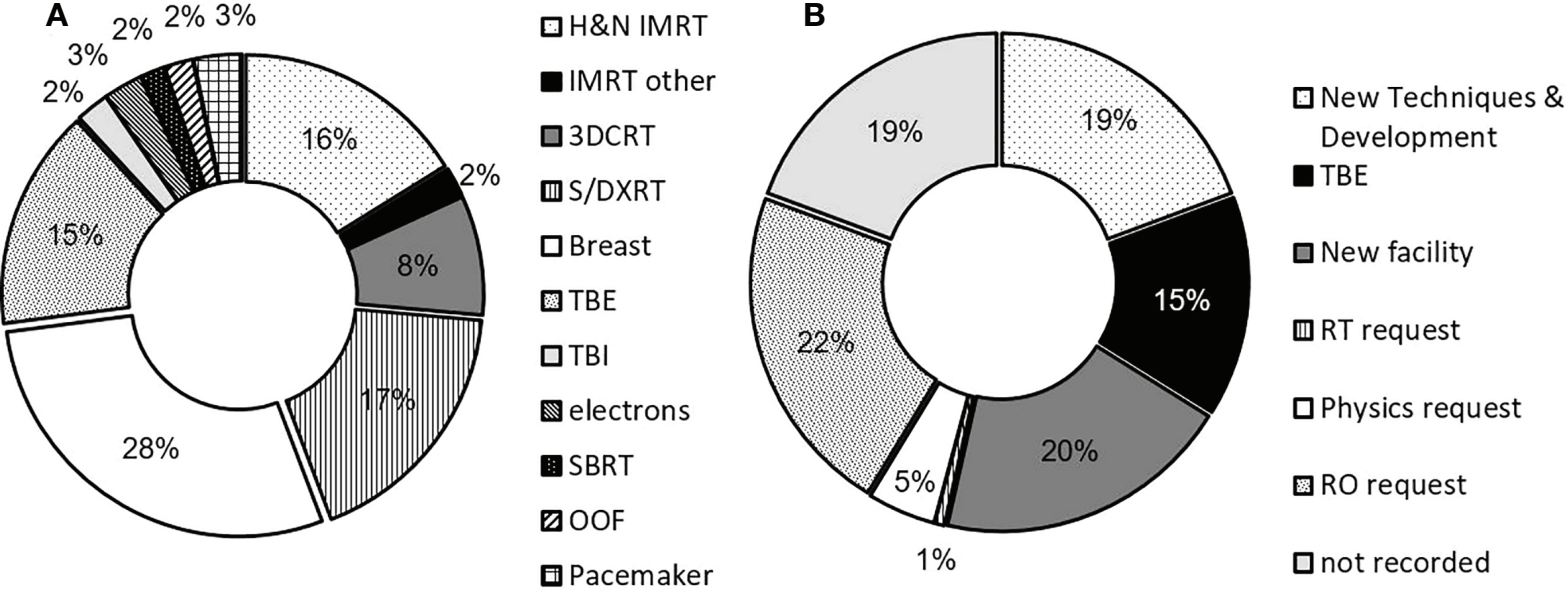
**(A)** Measurement applications for TLD service across all campuses and **(B)** distribution of TLD requests. Of the records where request data was available, radiation oncologists (ROs) were the highest instigator for TLD measurements, followed by opening of new radiotherapy facilities. Measurement applications include out-of-field (‘OOF’), stereotactic ablative body radiotherapy (‘SBRT’), conventional electron treatments (‘electron’), total body irradiation (‘TBI’), total body skin electron (‘TBE’), 3D-conformal radiotherapy (‘3DCRT’), breast, superficial and deep kilovoltage x-ray (‘S/DXRT’) treatments as well as IMRT. TBI is not routinely measured, and counts as a special request.

Treatment techniques for which TLD measurements were performed are shown in **Figure 3A**. Breast radiotherapy was the most common application, followed by superficial and deep kilovoltage x-ray (S/DXRT) treatments and head and neck (H&N) IMRT. All measurements were performed either by or under direct supervision of a radiation oncology medical physicist, with sufficient knowledge of TPS dose calculation algorithms and their limitations, TLD theory, and the physics of radiation transport and interactions. Data shown is collated for all four campuses where TLD measurements were performed. In addition, the original reasons given for each TLD measurement are summarized in **Figure 3B**. Requests from the treating RO were the most frequent background of patient TLD measurements, followed by opening of new facilities and new techniques and development, often requested and initiated by the medical physicists. On two occasions during the investigation our institution opened new radiotherapy facilities after a move. Under these circumstances the first patients treated with every radiation quality will – as far a reasonably achievable - have an *in vivo* dosimetry measurement performed which results in a relatively large number of measurements.

**Figure 4:**
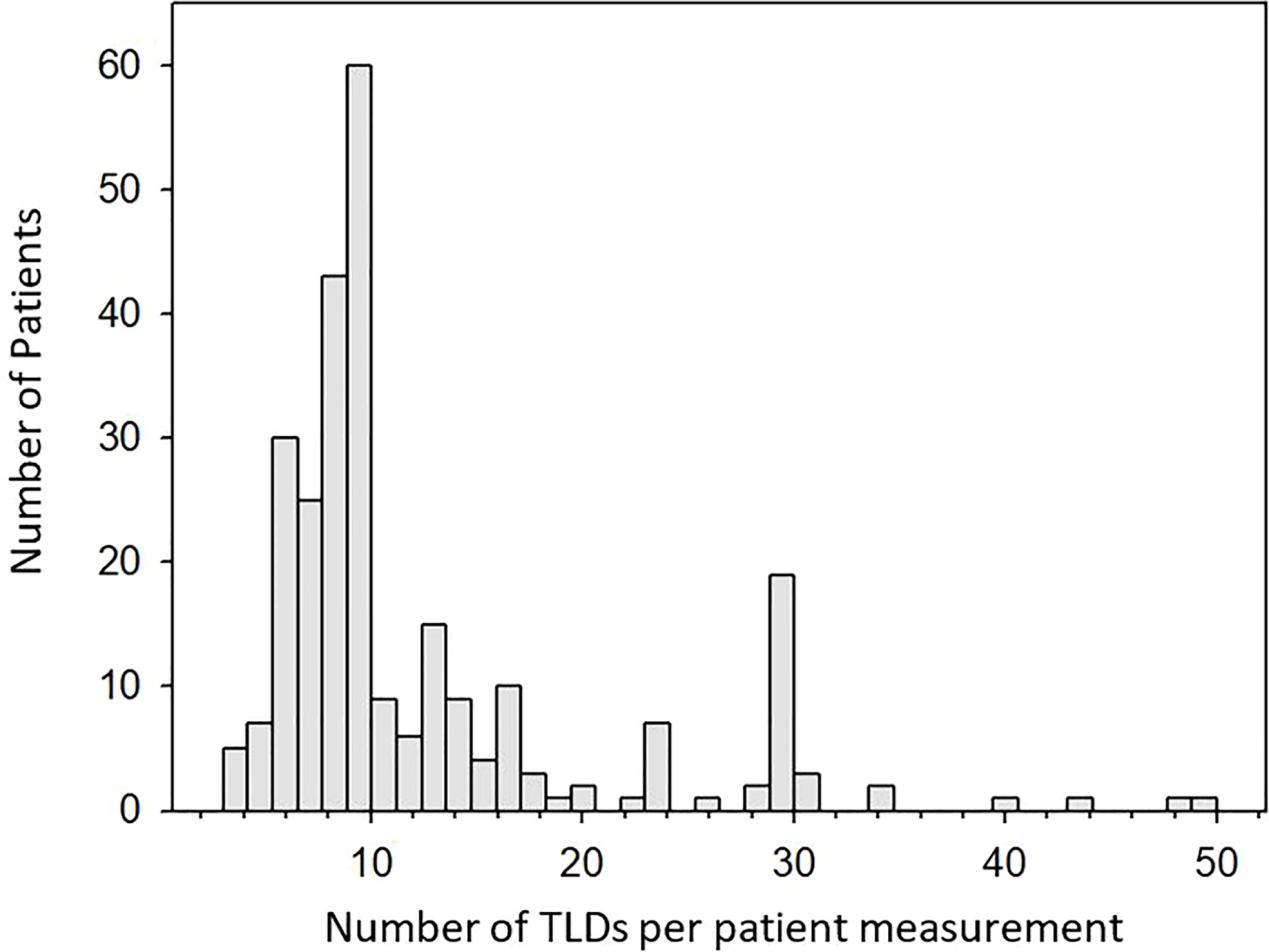
Histogram showing the number of TLDs used per patient measurement. Up to 10 TLDs are often used for conventional cases. The peak at 30 TLDs is a result of TBE treatments which in rare cases can also require up to 50 TLDs.

The number of TLD chips used per measurement is shown in **Figure 4**. TBE patients require measurements at 26 anatomical locations for two consecutive fractions for dose verification as per local practice, with additional measurement locations often requested by the treating RO resulting in up to 50 TLDs being used for a single measurement. Most TBE measurements require 28 TLDs, shown by the increased frequency in **Figure 4**. At least two TLDs were used for each measurement for any patient, with most measurements requiring between six and ten TLD chips, providing better measurement confidence than would be possible with a single dosimeter. The number of TLDs required for a given measurement was dependent on the nature of the measurement, with a larger number of TLDs often provided for cases where measurements were performed in the presence of dose gradients.

### Actionable findings

A total of 310 *in vivo* TLD measurements were performed over the two-year time period out of approximately 13 000 treatment courses delivered across all campuses. All TLD results were reviewed by a physicist who was responsible for interpretation of results and for following up any apparent discrepancies. Discrepancies were either accepted due to TPS limitations or exploratory-type TLD measurements, or actioned if no cause could be identified. The number of patient TLD measurements which resulted in a change to patient management are summarized in **Table 1**. Some 55 of these measurements were repeats for a particular patient, therefore 255 unique patient records were available for review. During that time a total of three major discrepancies between measured and expected dose were identified, equating to below 1% of all measurements performed. One such error lead to the purchase of new software to prevent further accidents – a change in clinical practice to improve patient safety brought on through *in vivo* dosimetry. As the *in vivo* measurements are targeted it is likely that these discrepancies are also indicative of the overall error rate which would be therefore of the order of 0.02 to 0.03% or 1 in 4000 treatment courses. This assumption however is based on other forms of quality assurance being able to catch any errors for patients who did not have *in vivo* dosimetry. As such, the true error rate may be higher.

**Table 1:** **–** Number of TLD measurement cases requiring intervention.

	Total (n)	% of measurements
Major discrepancies	3	<1
Discrepancies	24	8
Treatment change suggested based on TLDs	14	5

In an additional 8% of cases, a smaller discrepancy between measured and expected dose was found. A discrepancy was identified in the difference between measured and expected dose being greater than the measurement uncertainties associated with a particular TLD measurement. These measurement uncertainties for TLD are typically of the order of 5%. Discrepancies in this category were not classified as errors since an explanation for the discrepancy could be identified upon further review. In addition, fourteen instances of a request for a change to patient management were noted. These were initiated by the RO following review of TLD results. Again however, these were not due to treatment error or misadministration but reflect genuinely uncertain dose for example at skin or behind air cavities under bolus.

## Discussion

The current work summarizes the impact of an *in vivo* dosimetry programme using TLD across a large and complex radiotherapy department spread across multiple campuses treating approximately 7,000 patients annually. Three potentially serious incidents were identified during the two-year review period. In each case, discrepancies between total projected measured dose and planned dose to the region measured were in excess of 10%. Once a major discrepancy was identified, which due to the nature of *in vivo* dosimetry typically happens early in the treatment course, treatment plans were immediately corrected and remaining treatment fractions adjusted such that the total dose was delivered as prescribed. It is important to note that these errors were identified through *in vivo* dosimetry, after passing other quality assurance checks and - by the very nature of *in vivo* dosimetry - reaching the patient.

A change of practice was introduced following one of these incidents in particular, which involved an unusual two-field beam arrangement for a kilovoltage radiotherapy treatment. It was identified that in addition to the unusual field arrangement the lack of independent point-dose monitor unit calculation software was likely a contributing factor. Consequently, a computerized system has been purchased. The second error arose from manual data entry when treatment dose per fraction and number of treatment fractions had been inadvertently swapped as they were entered into the TPS, doubling the dose per fraction. The third error identified a region of overlap between a previously treated region and a new planning target volume. While the previous field was marked at CT using bolus, the bolus slipped out of position before the scan commenced. As a result, contours for the new planning target volume inadvertently encompassed a small region of the previous treatment region. TLD measurements within the overlap region identified the error and the plan was adjusted.

Several other differences were noted as summarized in **Table 1** but were not the result of treatment misadministration; rather, discrepancies in these cases could be explained either by TLD measurement conditions or known limitations of the TPS eg. in the build-up region. These cases required a physicist to review the treatment plan and related TLD results in greater detail. Dose discrepancies were common in cases where surface dose measurements were compared to the TPS dose calculations with no build-up material. Out-of-field doses were also at times in disagreement with the TPS, another known TPS limitation (13, 14).

*In vivo* dosimetry is a targeted activity where novel, complex and/or unusual treatment scenarios are preferentially measured. As our department uses several other quality control activities from record and verify systems to independent plan checking and patient specific QA only about 2 to 3% of patients undergo *in vivo* dosimetry. As such the true rate of major discrepancies per patient is significantly less than 0.1% (3 in 13 000) which is also in line with an earlier comprehensive evaluation of our incident reporting system (15).

**Figure 1A** shows that TLDs were used for a large range of dose measurements from low dose out-of-field measurements (mGy) to high dose palliative treatments (10 Gy single fraction). The response across this dose range for LiF : Mg,Cu,P is reported to be linear up to 10 Gy (16). Since a combination of factors can affect TLD response, the linearity of our system as a whole was verified during commissioning of the TLD service. The TLD system was found to be linear between 10 cGy to 10 Gy.

The TLD service was used for a wide range of applications which are summarized in **Figure 3**. The highest demand for TLD measurements were applications in breast radiotherapy, followed by superficial and deep therapies (S/DXRT), total body electron (TBE) and head and neck IMRT treatments. Skin reactions are often particularly important in these treatments and thus demand for TLD measurements was less for treatment techniques involving deep-seated targets. New techniques and development also placed high demand on the service with examples including the implementation of 3D-printed bolus as well as IMRT for breast using the Eclipse electronic compensator (17), for which additional *in vivo* dosimetry was employed when initially released for clinical use.

In some cases, despite agreement between TLD measured dose and expected dose, treatment plans were occasionally adjusted at the request of the ROs. Treatment changes were requested in 5% of cases as shown in **Table 1**. Adjustment of bolus during a radiotherapy treatment course after TLD confirmation of skin dose is one such example. Requests for treatment modification were most common in kilovoltage radiotherapy. These usually involved an adjustment of prescription to correct for a slight over- or under-dose within the treatment region involving an irregular surface. In such cases, while the dose measured in the field center was within 5%, doses at the field edge due to an irregular surface were different to field center, highlighted by TLDs in these regions, and an adjustment was made at the request of the RO. In the absence of a TPS for these treatments, *in vivo* dosimetry lends itself not only for ascertaining dose heterogeneity in-field but also reporting doses to nearby healthy tissue. While treatment was delivered accurately in these cases the *in vivo* measurements still had an impact on patient care.

Most of the *in vivo* dosimetry measurements are for individual patients; however, *in vivo* dosimetry is also a genuine quality improvement tool in particular in scenarios where quantitative outcomes are difficult to obtain otherwise. Total body skin irradiation and superficial treatments in the head and neck region are good examples for this. TLD is also useful in low-dose environments where the radiation quality is not well defined, for example area monitoring. The TLD service described herein is also used to monitor occupied areas around radiotherapy bunkers, particularly after a new machine is installed. The high sensitivity of LiF : MgCuP makes it a useful material for these scenarios, being able to detect radiation doses at near-background levels over several months without significant degradation of TL signal.

It is difficult to put a value on the TLD *in vivo* dosimetry service. However, without doubt the combination of major findings, albeit a small number, and minor adjustments add value to the radiotherapy process and provide additional confidence in treatment delivery. Given the cost of the service and the work load of a TLD based service we feel that the targeted approach where ROs, RTs and Medical Physicists can request measurements appears to be the most appropriate way forward.

## Conclusion

TLD was found to be an appropriate dosimeter for a wide range of *in vivo* measurement scenarios across multiple campuses of a large radiotherapy department. The linear dose response and relatively small energy dependence of LiF : Mg,Cu,P enabled measurements over a large clinical dose range, from mGy to several Gy. Treatment techniques include 3-D conformal radiotherapy and IMRT with megavoltage photons, megavoltage electron treatments and more complex treatments such as total body skin electron therapy and superficial and deep therapies in the kilovoltage range. Errors were found in less than 1% of measurements performed. Identification of these errors helped facilitate changes in practice for quality improvement. Additional resources are required to run a TLD service which should be factored in during commissioning. Interpretation of TLD measurements is not always straight forward as it is not always possible to compare a TLD result to a known dose at the same location. A skilled operator with sufficient knowledge of TPS dose calculation algorithms and their limitations, TLD theory, and the physics of radiation transport and interactions is required. TLD continues to be an important component of quality assurance procedures across the department.

## Data availability statement

The data analyzed in this study is subject to the following licenses/restrictions: De-identified patient dose records used for quality assurance, secured within the hospital network. No additional patient information was acquired above routine quality assurance tasks, conducted by clinical staff. Requests to access these datasets should be directed to Peta.Lonski@petermac.org.

## Ethics statement

Ethical review and approval was not required for the study on human participants in accordance with the local legislation and institutional requirements. Written informed consent from the patients was not required to participate in this study in accordance with the national legislation and the institutional requirements.

## Author contributions

All authors listed have made a substantial, direct, and intellectual contribution to the work and approved it for publication.

## Conflict of interest

The authors declare that the research was conducted in the absence of any commercial or financial relationships that could be construed as a potential conflict of interest.

## Publisher’s note

All claims expressed in this article are solely those of the authors and do not necessarily represent those of their affiliated organizations, or those of the publisher, the editors and the reviewers. Any product that may be evaluated in this article, or claim that may be made by its manufacturer, is not guaranteed or endorsed by the publisher.
